# Case report: Localized coloproctitis caused by novel *Basidiobolus arizonensis* in a dog

**DOI:** 10.3389/fvets.2024.1427496

**Published:** 2024-09-09

**Authors:** Annalise Black, Marcellina Wiertek, Sylvia Ferguson, Kathryn Wycislo, Laura Rayhel, Heather Reid, Nathan Wiederhold, Connie Cañete-Gibas

**Affiliations:** ^1^Department of Pathology & Microbiology, College of Veterinary Medicine, Midwestern University, Glendale, AZ, United States; ^2^College of Osteopathic Medicine, Midwestern University, Glendale, AZ, United States; ^3^Department of Specialty Medicine, College of Veterinary Medicine, Midwestern University, Glendale, AZ, United States; ^4^Department of Medicine, College of Veterinary Medicine, Tufts University, North Grafton, MA, United States; ^5^Fungus Testing Laboratory, Department of Pathology & Laboratory Medicine, University of Texas Health Science Center, San Antonio, TX, United States

**Keywords:** case report, fungal, basidiobolomycosis, zygomycosis, proctitis, inflammatory bowel disease (IBD), canine

## Abstract

A 6-year-old male neutered boxer mix canine was presented for a one-month history of dyschezia, hematochezia, and constipation. Colonoscopy and endoscopic biopsies revealed non-specific lymphoplasmacytic, eosinophilic colitis. Despite pursuing various therapies over a 3.5-month clinical course (including hypoallergenic diet, antibiotics, prokinetics, laxatives, and anti-inflammatory glucocorticoids), the patient’s condition did not improve. Two and a half months after initial presentation, the patient developed circumferential proctitis with multiple draining tracts and obstipation. Humane euthanasia and postmortem examination were elected. Gross and histological findings revealed locally extensive pyogranulomatous coloproctitis with many intralesional PAS-positive, GMS-negative 30–40 μm in diameter, hyaline, pauciseptate, irregularly branching fungal hyphae, hyphal bodies or chlamydospores and 25–45 μm in diameter thick-walled zygospores. Fungal culture of fluid from the draining tracts was performed, and DNA sequence analysis of the ITS and partial LSU of the nuclear ribosomal RNA genes were used to identify and confirm a novel species, *Basidiobolus arizonensis*. *Basidiobolus* spp. are saprobes in the order Basidiobolales and most commonly cause granulomatous infections of the skin, respiratory tract, and gastrointestinal tract in veterinary species and humans. To the authors’ knowledge, this is the first report of novel *Basidiobolus arizonensis* causing localized coloproctitis in a dog.

## Introduction

Basidiobolomycosis is a rare but emerging disease that primarily affects animals and people in tropical and subtropical climates. Originally, the genus *Basidiobolus* was primarily classified as an insect pathogen in the phylum Zygomycota. However, with the availability of molecular data, this taxonomic approach was challenged, and the phylum Zygomycota was found to be a polyphyletic group (1–4). The current taxonomy places *Basidiobolus* in the subkingdom Basidiobolomyceta, phylum Basidiobolomycota, order Basidiobolales and family Basidiobolaceae (3). There are currently eight phylogenetically confirmed species within this genus; *B. haptosporus*, *B. heterosporus*, *B. magnus*, *B. meristosporus*, *B. microsporus*, *B. minor*, *B. ranarum*, and *B. omanensis* (5). Though previously classified within the obsolete phylum Zygomycota and now within the phylum Basidiobolomycota, infections by this genus are still informally characterized under the broad term “zygomycosis” (6, 7). These fungi are found in soil, stagnant water, and as commensal microbes in the gastrointestinal tracts of some amphibians, fish, reptiles, and bats (8–10). Most cases in people have been reported in tropical and subtropical regions of Asia, South America, East and West Africa, and more recently in England, the southwestern United States, Saudi Arabia, Iran, and Iraq (11, 12). Host entry by fungal spores is assumed to occur though insect bites, minor trauma, inhalation, and ingestion (11). Infection commonly results in cutaneous, rhinonasal, pulmonary, and gastrointestinal disease (7, 11, 12). In both humans and animals, basidiobolomycosis often resembles other, more common diseases, which makes identification even more difficult (6, 13–18). Furthermore, once the fungus is identified, disease is often advanced and treatment is unpredictable due to the scarcity of successfully treated cases reported in the literature. This report discusses the clinicopathologic and molecular diagnostic findings in the case of a dog in Arizona with confirmed basidiobolomycosis attributable to novel species *Basidiobolus arizonensis.* To the authors’ knowledge, this is the first report of infection with this species in human and veterinary medical literature.

## Case description

A 6-year-old male neutered boxer mix canine was presented to Midwestern University Companion Animal Clinic Small Animal Internal Medicine (MWU CAC SAIM) department for a one-month history of dyschezia and intermittent hematochezia. The patient had no travel history outside of Arizona. Initial evaluation by the referring veterinarian showed significant constipation on abdominal radiographs but no other abnormalities. The referring veterinarian prescribed metoclopramide (0.2 mg/kg PO q 8 h), lactulose (0.2–0.4 mL/kg PO q 8 h), famotidine (0.9 mg/kg PO q 12 h) and sucralfate (32 mg/kg PO q 8 h). A probiotic (Proviable Forte) and canned pumpkin were also recommended for ongoing use. Initial physical exam at MWU CAC SAIM revealed a body condition score of 9/9, mild dental disease, and sinus arrhythmia. On digital rectal exam, there was a 1.5 cm in diameter, indistinct, soft nodule palpated on the ventral rectal mucosa, approximately 5.0 cm orad to the anus. Complete blood count and serum biochemistry panel were performed and revealed mild lymphopenia (564/uL, reference interval (RI) 690-4500/uL), moderate eosinophilia (4,512/uL, RI 0-1200/uL) and basophilia (282/uL, RI 0-150/uL), and hyperproteinemia (7.8 g/dL, RI 5–7.4 g/dL) characterized by mild hyperglobulinemia (4.0 g/dL, RI 1.6–3.6 g/dL). Abdominal ultrasound revealed diffuse mild thickening of the ventral colonic wall (0.26 cm) with preserved layering. Just craniad to the pubic bone, a focal nodule was indistinctly visualized in the ventral colonic wall (1.24 cm). The dorsal wall of the colon could not be evaluated due to the presence of gas in the colonic lumen. Both medial iliac lymph nodes were mildly enlarged (left: 2.21 × 1.28 cm, right: 1.89 × 1.31 cm).

On ileocolonoscopy, most of the mucosa was unremarkable, other than a few erosions within the transverse colonic mucosa. The palpated rectal nodule was not visualized on colonoscopy. There was focal hemorrhage on the ileocecal valve mucosa. Biopsies of this lesion, as well as general mucosal pinch biopsies from the colon and ileum, were taken for histopathology. A touch prep cytology of the ileocolic sphincter biopsy was performed and revealed moderate mixed inflammation with bacteria, chronic hemorrhage, and structures most consistent with degenerating intestinal epithelial cells. Histopathology of the ileocecal valve revealed moderate, acute superficial mucosal hemorrhage admixed with gram-positive bacilli. Partial thickness samples of the ileal and colonic mucosa showed moderate, chronic, multifocal, eosinophilic, lymphoplasmacytic ileocolitis with mild colonic crypt hyperplasia. Other than the bacilli noted in the sample from the ileocecal valve, no etiologic agents were identified on histopathology. Periodic acid-Schiff (PAS) staining of the colon and cecocolic junction was unremarkable. There was no evidence of histiocytic ulcerative colitis, pyogranulomatous inflammation, or neoplasia. The patient was treated with a single dose of praziquantel/pyrantel pamoate/febantel (Drontal Plus (TM) Large Dog Tablets, 136 mg, 1 and ½ tablets). Psyllium husk supplementation was initiated, and probiotic treatment was continued. Lactulose and metoclopramide were discontinued. A strict hypoallergenic diet trial was initiated (Royal Canin Hydrolyzed Protein).

**Figure 1:**
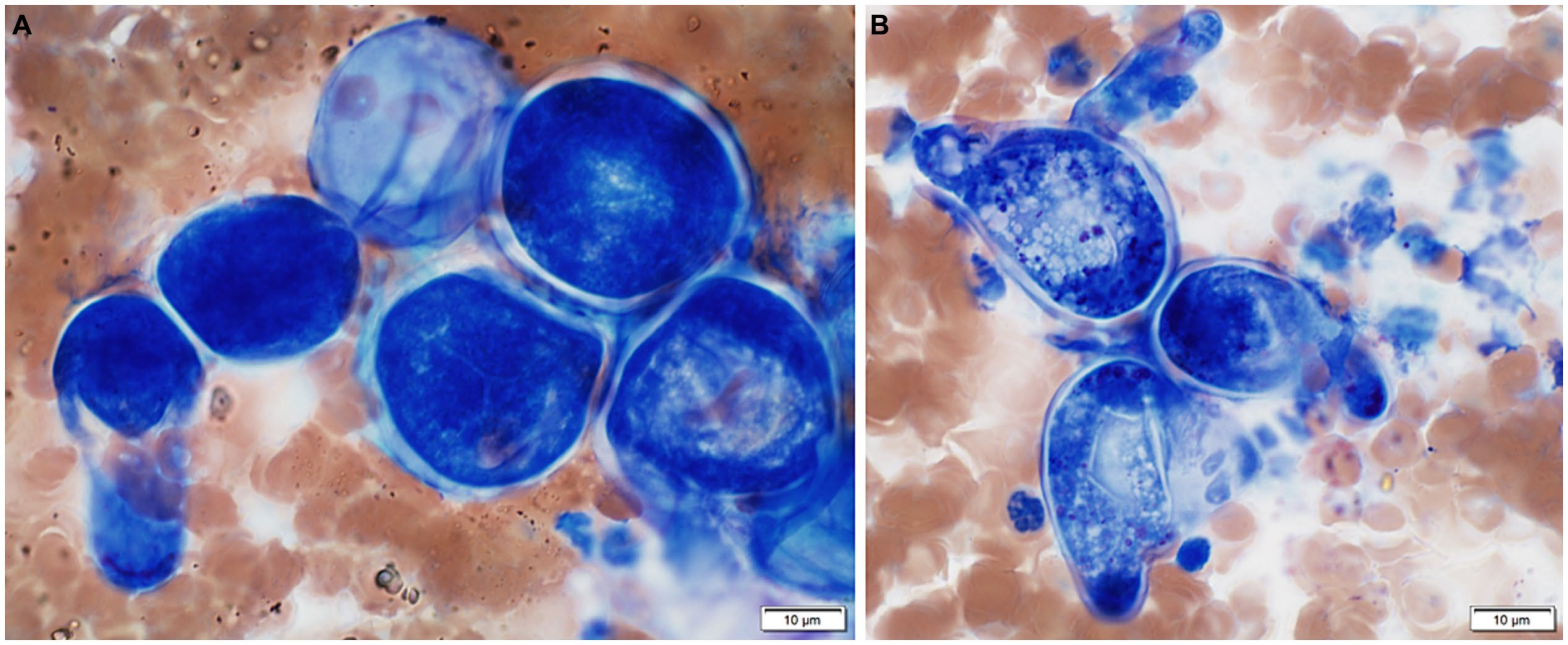
Cytologic features from a fine needle aspirate of thickened perianal tissue in a boxer mix canine with basidiobolomycosis. **(A)** Several deeply basophilic hyphal bodies or chlamydospores. Wright-Giemsa, 100x. **(B)** Basophilic conidia with lateral papillae. Wright-Giemsa, 100x.

Approximately 1 month after initiating the diet trial, the owner indicated a lack of clinical response, with continued dyschezia and soft stools. The patient was started on enrofloxacin (12.1 mg/kg PO q 24 h) and the hypoallergenic diet was continued. One month later, no clinical improvement was observed. However, when the owners attempted to feed a commercial diet, the patient’s tenesmus and hematochezia worsened. The hypoallergenic diet was reimplemented, and the patient was started on prednisone (0.75 mg/kg PO q 24 h). Six days after initiating prednisone, the referring veterinarian examined the patient due to acute worsening in clinical signs and severe, circumferential anal thickening. Eleven days after initiating prednisone, the patient was reevaluated at MWU CAC for inability to defecate for multiple days, ongoing tenesmus, and licking the perineum. Severe circumferential tumefaction, erythema, and erosion of the anorectal and perianal tissues extended approximately 2.5 cm into the rectum, with draining serosanguineous exudate. Lactulose (0.4 mL/kg q 8–12 h) was reinitiated and the patient was discharged overnight. Fine needle aspiration of the affected site revealed chronic suppurative to pyogranulomatous inflammation with numerous 20–60 μm in diameter, round, deeply basophilic hyphal bodies or chlamydospores and fewer 8–10 μm in diameter, deeply basophilic, broad, pauciseptate hyphae. Similar structures, consistent with conidia, occasionally contained lateral papillae (Figure 1). Initial differential diagnoses considered were mucormycosis, entomophthoromycosis, and phaeohyphomycosis.

The patient was represented the following day after vomiting multiple times overnight and worsening perianal swelling. Samples from the anal lesion aspirates were sent to the University of Florida (UF) Molecular Fungal ID Lab for panfungal polymerase chain reaction (PCR) and to MWU Clinical Microbiology Laboratory for fungal culture. An empirical treatment plan was created while awaiting results. The patient was to start oral maropitant, itraconazole, and terbinafine and return the next morning to receive his first of 10 planned intravenous injections of amphotericin B lipid complex. The prednisone was tapered to 0.26 mg/kg/d PO with intention to discontinue. However, the patient rapidly declined overnight, and the owners elected to humanely euthanize.

**Figure 2:**
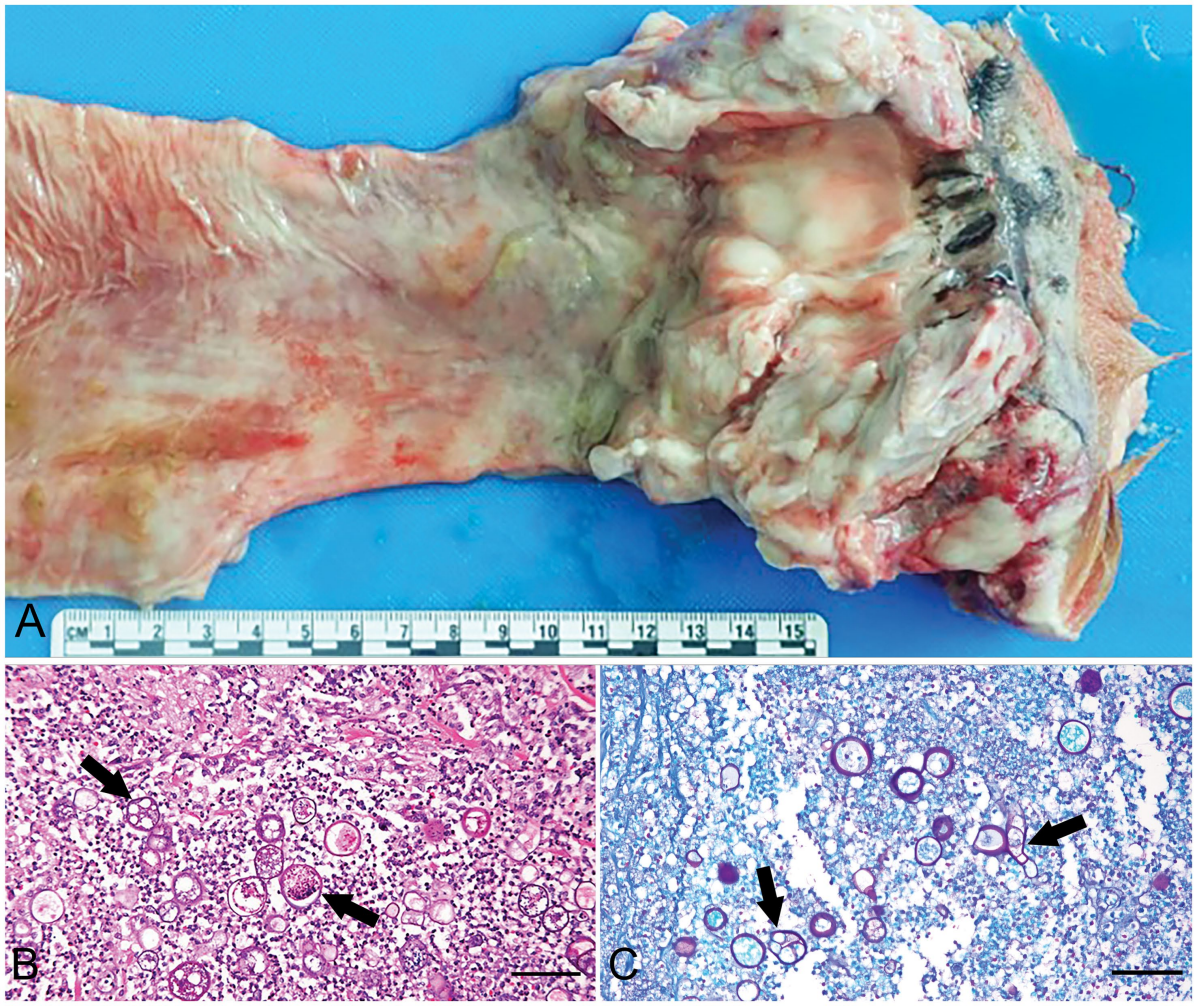
Gross and microscopic features of colorectal basidiobolomycosis in a boxer mix canine. **(A)** Rectum and colon on cut section. The rectum and most aborad colon are circumferentially thickened. **(B)** Photomicrograph of rectum demonstrates areas of thickening are the result of pyogranulomatous inflammation centered on numerous fungal organisms that often form 25–45 μm in diameter zygospores with a thick eosinophilic wall (arrows) H & E. Scale bar = 75 μm. **(C)** Fungal organisms are strongly Periodic Acid Schiff (PAS) positive (arrows). Scale bar = 75 μm.

On postmortem examination, perianal soft tissue and rectum were markedly thickened up to 5.0 cm circumferentially by multifocal to coalescing, tan-white, firm nodules (Figure 2A). The nodules extended approximately 2.0 cm into the adjacent colon. There were multifocal mucosal ulcerations throughout the distal colon, rectum, and anus. There were multiple cutaneous fistulous draining tracts within and surrounding the anus. The sublumbar and inguinal lymph nodes were moderately to markedly enlarged and firm. The ileocecal valve was moderately, circumferentially thickened and mottled tan to pink. The colon was severely gas distended, hyperemic with multifocal mucosal hemorrhages, and contained a large amount of soft feces. Representative samples were collected and placed in 10% neutral-buffered formalin. Tissue samples were routinely processed and stained with hematoxylin and eosin (H&E).

On histopathology from the distal colon and rectum, there was severe, chronic-active, regionally extensive pyogranulomatous colitis and proctitis with intralesional Gomori Methenamine Silver (GMS) negative (Figure 2B), Periodic acid-Schiff (PAS) positive (Figure 2C) fungal hyphae and hyphal bodies or chlamydospores. Hyphae were 30–40 μm in diameter, thin-walled, hyaline, pauciseptate, bulbous, and irregular. Zygospores were 25–45 μm in diameter with a thick eosinophilic wall, foamy amphophilic cytoplasm, and central nucleus with a prominent basophilic nucleolus. Fungal hyphae were sometimes surrounded by a hypereosinophilic sheath (Splendore-Hoeppli phenomenon). Fungal organisms occasionally invaded vessel walls and lumina. A regional lymph node was moderately hyperplastic and reactive. The medullary and subcapsular sinuses were mildly expanded by histiocytes, plasma cells, erythrocytes, erythrophages, and hemosiderophages.

Consistent with the previous biopsies, there was chronic-active eosinophilic, lymphoplasmacytic colitis. Gram staining showed small numbers of gram-positive cocci and bacilli, and fewer gram-negative bacilli at the luminal surface. Histopathology of the small intestine revealed that the lamina propria was moderately expanded by plasma cells, lymphocytes, and fewer eosinophils, which extended into the muscularis mucosae. Although the pancreas was grossly unremarkable, microscopically there was mild, acute pancreatitis.

Fungal culture of fluid exuding from the perianal soft tissue was positive for growth at 15 days on Sabouraud Chloramphenicol Gentamicin Dextrose, Inhibitory Mold, Mycosel, and Sabouraud Dextrose (Emmons) agars. Identification of the organism via MALDI-TOF MS was not possible. The perianal cytology slide was sent to the University of Florida Molecular Fungal ID Lab for panfungal PCR. Targeting the internal transcribed spacer (ITS) region, agarose gel electrophoresis yielded two bands of DNA at approximately 300 and 350 bp. The DNA was purified from the gel and sequenced. The resulting 350 bp contig sequence was analyzed with NCBI BLAST (https://blast.ncbi.nlm.nih.gov/Blast.cgi). The sequence matched *Basidiobolus ranarum* with 97.69% identity.

A pure isolate, UTHSCSA DI22-113, was then sent to the Fungus Testing Laboratory, University of Texas Health Science Center at San Antonio (FTL) and was subcultured onto potato flakes agar (PFA, prepared inhouse) and Sabouraud Dextrose agar (SDA). Growth temperatures were assessed at 25, 37 and 40°C. Wet mounts and slide culture preparations were also done for microscopic observation. Mycelia were harvested from a week old PFA culture for DNA sequencing to confirm the species following previously described methods (13). PCR and sequencing primers for ITS (forward BMB-CR/reverse ITS4) and LSU (forward NL1/reverse NL4) genes were used (Vilgalys lab, Duke University, updated Feb 3, 1992; https://sites.duke.edu/vilgalyslab/rdna_primers_for_fungi) (14). BLASTn searches were performed using the ITS and LSU sequences in GenBank and phylogenetic analyses using maximum likelihood were conducted in IQ-Tree (15, 16). Substitution models for maximum likelihood analysis were determined using the ModelFinder and the Akaike information criterion, and branch support was evaluated using 1,000 resampling of ultrafast bootstrapping (UFBoot), the Bayesian-like modification of the aLRT (aBayes) and the aLRT with nonparametric Shimodaira-Hasegawa correction (SH-aLRT), all of which are implemented in IQ-Tree (17–19).

**Figure 3:**
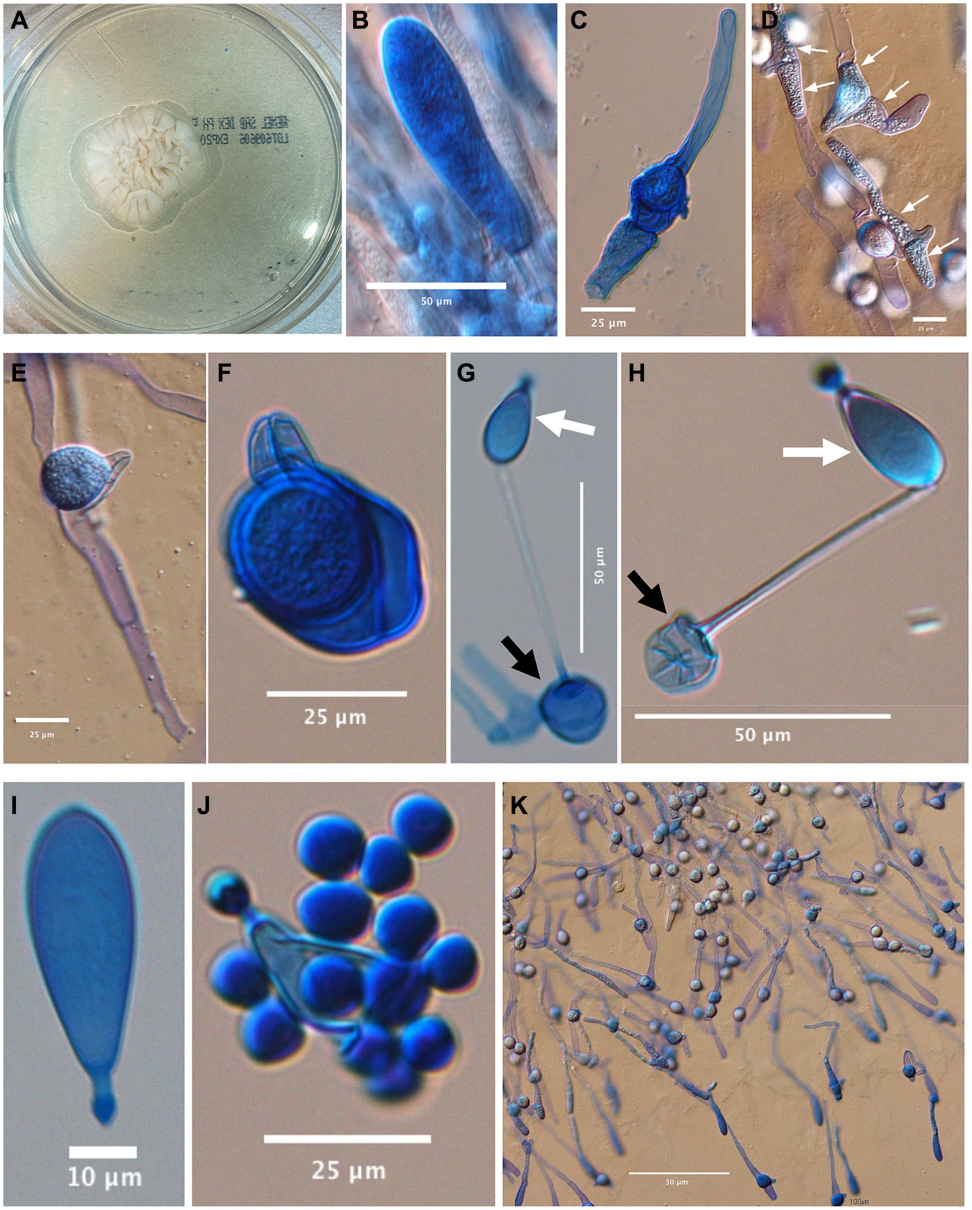
*Basidiobolus arizonensis.* **(A)** White to cream, rugose colony on Sabouraud dextrose agar, 7d, 25°C; microscopic images from a 6d slide culture at 25°C on PFA: **(B)** distal portion of elongating hypha at margin of growing mycelia. **(C,D)** Encounter of opposite sex hyphae before the formation of lateral beaks (white arrows). **(E,F)** After the exchange of genetic material, zygospores develop with their characteristic beak. **(G,H)** Globose conidia (black arrow) bearing a uninucleated adhesive conidium (white arrow). **(I)** Detached adhesive conidium. **(J)** Adhesive conidium converted to sporangium releasing globose sporangiospores. **(K)** Germinating zygospores.

**Figure 4:**
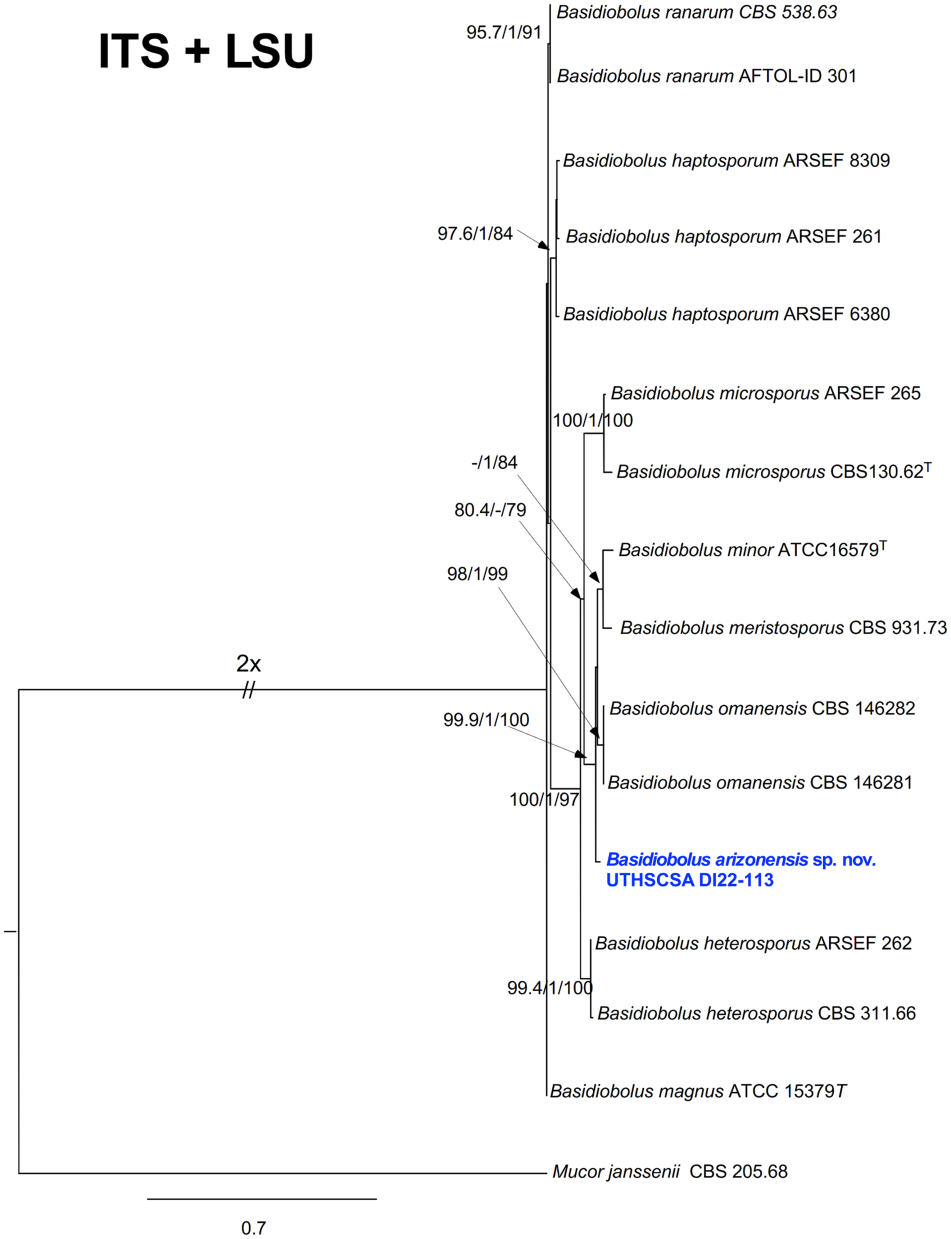
Maximum likelihood phylogram generated from combined ITS and partial LSU sequences of UTHSCSA DI22-113 and representative *Basidiobolus* spp. Numbers on the nodes are (SH-aLRT) ≥ 80%; (aBayes) ≥ 0.95; (UFBoot) ≥ 75%. The new species proposed is indicated in bold blue. T = ex-types of strains. The tree is rooted to *Mucor janssenii* CBS 205.68^T^.

The colonies on PFA were subhyaline, glabrous or waxy, and without aerial mycelium. The wet mount and slide culture preparations showed morphological characteristics consistent with *Basidiobolus* by the production of zygospores with paired protuberances or lateral beaks leading to a structure known as a “beaked zygospore” (Figure 3). Generally, distinct morphological traits are minimal or absent between *Basidiobolus* species. BLASTn searches (https://blast.ncbi.nlm.nih.gov/Blast.cgi accessed on December 7, 2022 and again on March 9, 2023) of the ITS and partial LSU sequences of the isolate (UTHSCSA DI22-113) showed that the ITS sequence matched *B. meristosporus* CBS 931.73^T^ with 93.94% similarity, *B. meristosporus* CBS 140.55 at 94.26%, followed by *B. omanensis* CBS 146281^T^ at 94.10%, while LSU top matches were *B. omanensis* CBS 146281^T^ at 98.40%, *B. meristosporus* ATCC 14450 at 98.29%, *B. meristosporus* CBS 931.73^T^ at 97.24, *B. meristosporus* CBS 140.55 at 98.29% and some unnamed *Basidiobolus* species F43-5 and F15-1 both at 98.43%. Substitution models used for maximum likelihood were HKY + F + I + G4 (ITS), TN + F + I + G4 (LSU) and TN + F + I + G4 for combined ITS and LSU. BLASTn results and phylogenetic analyses of individual (not shown) and combined ITS and LSU sequences using the backbone tree of Al-Hatmi et al. (5), showed that UTHSCSA DI22-113 is closely related to but phylogenetically distant from the recognized species *B. meristosporus* and *B. omanensis* (Figure 4) (5). Species delimitation is difficult in the genus *Basidiobolus* because the genus comprises species that are morphologically similar. The genealogical concordance phylogenetic species recognition (GCPSR) is used to compare more than one individual gene genealogies to identify incongruences (20). Disagreement between gene genealogies is recognized as point of genetic isolation and species limits. Including a gene with incongruences could mask true evolutionary relationships among closely related species. Following the GCPSR criteria, where UTHSCSA DI22-113 is shown as a distinct lineage in the ITS and LSU individual and combined phylogenetic trees, we describe the novel species *Basidiobolus arizonensis.*

### Taxonomy

*Basidiobolus arizonensis* C. F. Cañete-Gibas, N. P. Wiederhold, A. Black, K. Wycislo, & C. Sanders sp. nov. (Figure 3).

Mycobank: MB847849.

Eukaryota; Fungi; Basidiobolomyceta; Basidiobolomycota; Basidiobolomycotina; Basidiobolomycetes; Basidiobolales; Basidiobolaceae; *Basidiobolus*.

Etymology: “arizonensis” refers to the state of Arizona in the United States of America, where the fungus was first isolated.

Typus: United States of America, Glendale, Arizona, isolated from fluid collected from draining tracts of the perineum in a dog; Collected on September 20, 2022 by Laura Rayhel and isolated on October 9, 2022 by Catherine Cruz and Ogi Okwumabua; Holotype = CBS H-25225; Ex-type = UTHSCSA DI22-113 = CBS 149860; *ITS*  = OQ449342; *LSU* = OQ450351.

Description: on SDA, 7d at 25°C, colonies grew 24–28 mm × 21 mm–27 mm in diameter, white to cream, glabrous, rugose and radially folded towards the rim (Figure 3A). Hyphal elements hyaline, coenocytic, occasionally septate (Figure 3E). Globose conidium, 18.6 mm – 18.78 mm diameter (black arrow) bearing adhesive conidium (white arrow) (Figures 3G,H). Encounter of opposite sex hyphae before the formation of lateral beaks (Figures 3C,D, white arrows). Mature zygospores, 21.4 mm – 27.88 mm in diameter with paired protuberances ready to germinate (Figures 3E,F,K). Detached adhesive conidium 14.94 mm–34.33 mm (L) × 12.27 mm–22.31 mm (W) (Figure 3I). Adhesive conidium converted to sporangium releasing globose sporangiospores, 7.2 mm-10.39 mm diameter (Figure 3J). Germinating zygospores (Figure 3K). Grows at 25, 37 and 40°C.

## Discussion

Basidiobolomycosis is a rare but emerging disease that has been reported in humans, dogs, and horses (21). In dogs, the few reported cases most commonly exhibit cutaneous (22), pulmonary (22, 23), and gastrointestinal disease (6, 13, 23, 24). Specifically, canine gastrointestinal basidiobolomycosis has been reported in the stomach and small intestine (23), diffusely in the colon (24), and localized to the colorectal region (6). The present case was a localized, regionally extensive infection of the aborad colon, rectum, anus, and perineum, with a novel intralesional fungal species, *Basidiobolus arizonensis*. As the infection was confined to one region and involved the perineal skin, the most likely route considered is percutaneous inoculation. The present case is most similar in clinical history and affected site to Marclay et al. (6), which describes a 7-year-old castrated male French Bulldog with 1 month history of large intestinal diarrhea, tenesmus, hematochezia, and decreased activity (6).

This patient’s dyschezia, hematochezia, and constipation did not improve despite various conventional therapies for chronic inflammatory enteropathy initiated over a period of 3.5 months. The development of rectoanal swelling and worsening clinical signs temporally correlate to when anti-inflammatory glucocorticoids were initiated. Considering this association and postmortem examination findings, the ventral mucosal nodule identified on palpation and ultrasound early on in this patient’s clinical course may have represented *B. arizonensis* infection. On postmortem examination, the colonic region of infection predominantly involved the submucosa, muscularis, and serosa, sparing the mucosa. This could explain why pyogranulomatous inflammation and/or fungal organisms were not observed on the initial partial thickness endoscopic biopsies. Consequently, it’s plausible this infection was the underlying cause of the patient’s initial clinical signs and could have been exacerbated by the administration of anti-inflammatory glucocorticoids. Furthermore, underlying basidiobolomycosis in the colorectal region may explain why this patient’s condition was refractory to conventional therapies for common causes of canine chronic enteropathy. This patient also had concurrent mild to moderate, eosinophilic, lymphoplasmacytic enterocolitis. Though unlikely to completely account for the patient’s severe clinical signs, chronic enterocolitis likely contributed. Similar to the case presented herein, pythiosis often results in inflammation centered on the submucosal and muscular layers of the gastrointestinal tract (25). For this reason, surgical exploration and full-thickness biopsies are recommended (13, 25, 26). The mild, acute pancreatitis presumably also contributed to the patient’s worsening clinical signs and the development of vomiting.

The planned treatment course included trimodal antifungal therapies (itraconazole, terbinafine, and amphotericin B lipid complex). This approach was adapted from the treatments administered to the French Bulldog in the case report by Marclay et al. (6), with the addition of amphotericin B as a rescue treatment due to the patient’s severe clinical signs and inability to defecate. The cited paper is the only description of partially successful treatment of basidiobolomycosis in dogs in the veterinary literature. At the time the treatment course was planned, the etiologic organism’s genus was not definitively known.

In humans, basidiobolomycosis is reported in both immunocompetent and immunocompromised hosts (5, 27). However, a reason for susceptibility to *Basidiobolus* infection is typically only identifiable in the immunocompromised (28–30). In the existing literature on cases of canine basidiobolomycosis, immune status is largely not discussed. Parambeth et al. (13) mention the possibility that treatment failure in their case could have been due to unidentified immunosuppression. Grooters (25) asserts that animals with basidiobolomycosis are typically immunocompetent. Though immune status cannot be fully assessed from postmortem examination, there was no evidence of immunosuppression in this case aside from the administration of exogenous glucocorticoids.

The diagnosis of *Basidiobolus* infection is typically accomplished by culture of the fungus as the gold standard (31). However, identification to the species level is difficult by morphology alone. Proteomic and molecular techniques, such as MALDI-TOF and DNA sequence analysis, combined with phenotypic characterization are necessary for accurate species identification in this genus. In the present case, *B. arizonensis* grew on multiple agars, but identification by MALDI-TOF was not possible, even to a genus level. This is likely due to *Basidiobolus* spp. not being included in the database, as these infections are uncommon (personal communication – Okwumabua). The diagnosis of *Basidiobolus* infection is further complicated by its similar morphology to *Conidiobolus* spp., *Pythium* spp., and *Lagenidium* spp. on histopathology (25). Often, a combination of culture, PCR, histopathology, and/or immunohistochemistry are needed for a definitive diagnosis (13). Furthermore, *Basidiobolus* spp. are widely reported to be GMS-positive, so the lack of GMS staining in this species is confounding. The tissues were processed using xylene-free methods, and we speculate this could have interfered with GMS staining, although this is not a known complication of xylene alternatives. If basidiobolomycosis is strongly suspected, use of both GMS and PAS staining may be warranted.

## Conclusion

Cytology, histopathology, culture, and DNA sequence analysis of the fungus support this case as the first documented infection with *Basidiobolus arizonensis* in human and veterinary medical literature. Fungal coloproctitis is the presumptive cause of the underlying cause of chronic dyschezia and constipation in this patient, and the infection was likely exacerbated by anti-inflammatory glucocorticoids.

## Data Availability

The datasets presented in this study can be found in online repositories. The names of the repository/repositories and accession number(s) can be found in the article/supplementary material.
